# The *CfMK1* Gene Regulates Reproduction, Appressorium Formation, and Pathogenesis in a Pear Anthracnose-Causing Fungus

**DOI:** 10.3390/jof8010077

**Published:** 2022-01-14

**Authors:** Chaohui Li, Weibo Sun, Shulin Cao, Rongxian Hou, Xiaogang Li, Liang Ming, Jialiang Kan, Yancun Zhao, Fengquan Liu

**Affiliations:** 1 Institute of Plant Protection, Jiangsu Key Laboratory for Food Quality and Safety-State Key Laboratory Cultivation Base of Ministry of Science and Technology, Jiangsu Academy of Agricultural Sciences, Nanjing 210014, China; chaohuili@jaas.ac.cn (C.L.); 20170019@jaas.ac.cn (W.S.); caoshulin@jaas.ac.cn (S.C.); 2019202002@stu.njau.edu.cn (R.H.); 20040006@jaas.ac.cn (L.M.); 2 Institute of Pomology, Jiangsu Academy of Agricultural Sciences, Nanjing 210014, China; 20000003@jaas.ac.cn (X.L.); 201800701@jaas.ac.cn (J.K.)

**Keywords:** pear anthracnose, *Colletotrichum*, virulence, RNA-seq

## Abstract

*Colletotrichum fructicola*, the causal agent of pear anthracnose, causes significant annual economic losses. Mitogen-activated protein kinase (MAPK) cascades are highly conserved signal transduction pathways that play a crucial role in mediating cellular responses to environmental and host signals in plant pathogenic fungi. In this study, we identified an ortholog of the *FUS3/KSS1*-related MAPK gene, *CfMK1*, and characterized its function in *C. fructicola*. The *Cfmk1* deletion mutants exhibited poorly developed aerial hyphae, autolysis, no conidial mass or perithecia on solid plates. However, the conidiation of the *Cfmk1* mutant in PDB liquid medium was normal compared with that of the wild type (WT). Conidia of the *Cfmk1* mutant exhibited a reduced germination rate on glass slides or plant surfaces. The *Cfmk1* deletion mutants were unable to form appressoria and lost the capacity to penetrate plant epidermal cells. The ability of the *Cfmk1* mutants to infect pear leaves and fruit was severely reduced. Moreover, RNA sequencing (RNA-seq) analysis of the WT and *Cfmk1* mutant was performed, and the results revealed 1886 upregulated and 1554 downregulated differentially expressed genes (DEGs) in the mutant. The DEGs were significantly enriched in cell wall and pathogenesis terms, which was consistent with the defects of the *Cfmk1* mutant in cell wall integrity and plant infection. Overall, our data demonstrate that *CfMK1* plays critical roles in the regulation of aerial hyphal growth, asexual and sexual reproduction, autolysis, appressorium formation, and pathogenicity.

## 1. Introduction

Pear is one of the most consumed fruits in the global market and is widely cultivated, especially in China, Japan, and Korea [1]. Pear anthracnose caused by *Colletotrichum* fungi is an extremely destructive disease that causes serious economic loss in eastern Asia. Sudden and severe outbreaks of pear anthracnose occur in eastern Asia every few years [2,3,4]. In 2008, pear anthracnose spread rapidly across ~40,000 ha of orchards in Dangshan County of Anhui Province in China, causing economic losses of approximately 150 million dollars [5]. To date, studies on pear anthracnose have extensively focused on the biological characteristics and taxonomy of the pathogens, disease epidemiology, and disease management by fungicides or biocontrol agents [2,3,4,5,6,7]. Twelve species of *Colletotrichum* have been reported to infect cultivated pears in China [8]. The complexity of the species composition and the genetic differences among the populations make it difficult to study the molecular mechanism underlying the pathogenesis of pear anthracnose. Signal transduction pathways in pathogenic fungal cells are crucial for successful disease establishment [9]. In view of this, analyzing conserved signaling pathways in the dominant causal agent of pear anthracnose will facilitate uncovering its pathogenesis mechanism.

*Colletotrichum fructicola* is the dominant, and one of the most aggressive, species causing pear anthracnose disease in China [8]. The fungus is spread throughout fields via rain-splash dispersal of conidia and air transmission of ascospores from the sexual morph [10]. The occurrence of disease begins with spores landing on plant surfaces and germinating to form germ tubes and appressorium, followed by turgor-driven penetration of the cuticle and invasive growth in plant tissue [10]. In eukaryotic organisms, mitogen-activated protein kinases (MAPKs) play major roles in sensing extracellular signals and regulating various cellular processes, including cell growth, proliferation, differentiation and stress response. The mitogen-activated kinase Fus3/Kss1 is the downstream kinase in the MAPK signal transduction modules regulating the activation/repression of the mating and filamentation pathways in *Saccharomyces cerevisiae* [11]. In pathogenic fungi, its homolog was first reported to be necessary for appressorium formation and pathogenicity in *Magnaporthe oryzae* [12]. Subsequent studies of over 20 plant pathogenic fungi, including *Ustilago maydis* [13], *Colletotrichum gloeosporioides* [14], *Cytospora chrysosperma* [15], *Valsa mali* [16], *Fusarium oxysporum* [17], and *Alternaria brassicicola* [18], indicated that the Fus3/Kss1 MAPK pathway is highly conserved in fungal pathogens for the regulation of the plant infection process [19]. RNAi-mediated silencing of MAPK coding genes in *F. oxysporum* reduced its virulence to tomato plants [20]. Application of exogenous dsRNA could silence the MAPK Bmp3 in *Botrytis cinerea* and, thus, affected its fungal growth and pathogenicity in *Lactuca sativa* [21]. These studies suggest that MAPK signaling genes can potentially be targeted for broad-spectrum disease control. Understanding the function of Fus3/Kss1 MAPK signaling pathways in *C. fructicola* might provide theoretical evidence for developing novel and sustainable disease management strategies against pear anthracnose.

Although Fus3/Kss1 MAPK has a conserved role in pathogenesis, its functions in other developmental processes vary among different phytopathogenic fungi [19]. For example, in *Colletotrichum lagenarium*, knockout of the Fus3 ortholog had no obvious effects on mycelial growth, while in *Colletotrichum higginsianum* and *Colletotrichum truncatum,* the deletion mutants were attenuated for vegetative growth [22,23,24]. In *Fusarium graminearum*, disruption of the *gpmk1* gene resulted in mutants that exhibited reduced conidial production and were sexually sterile [25], while in *Fusarium verticillioides,* the mutant showed normal sexual reproduction and increased production of macroconidia [26]. While this MAPK is important for conidium germination in *C. lagenarium*, its ortholog regulates pycnidium formation and sclerotium development in *Mycosphaerella graminicola* and *Sclerotinia sclerotiorum* [19]. In *Alternaria alternata*, AsFus3 is important for copper fungicide resistance [27]. In *C. higginsianum* and *V. mali*, the Fus3 ortholog is implicated in the maintenance of cell wall integrity. In *F. graminearum and F. verticillioides,* the Fus3 ortholog is important for the biosynthesis of secondary metabolite toxins [26].

To promote the study of the pathogenic mechanism of pear anthracnose, in this study, we identified and characterized the functions of CfMK1, an ortholog of Fus3/Kss1 MAP kinase in *C. fructicola*. Here, we demonstrate that CfMK1 plays critical roles in appressorium formation, cell wall integrity, conidiation or sexual reproduction on solid medium, and in pathogenicity on pear leaves and fruit. Furthermore, RNA sequencing (RNA-seq) analysis performed in this study revealed that CfMK1 regulates the expression levels of several subsets of genes associated with the fungal cell wall and pathogenesis.

## 2. Materials and Methods

### 2.1. Fungal Strains and Culture Conditions

The *C. fructicola* WT strain NC40 was isolated from pear orchards in Jiangxi Province, China. Its species identity was confirmed by multilocus phylogenetic analyses (unpublished data). The wild type strain and all transformants used in this study were routinely cultured on PDA or CM plates at 28 °C as described previously [6]. For *C. fructicola* transformation, protoplast preparation and polyethylene glycol (PEG)-mediated transformation were performed as described previously [28]. For fungal DNA isolation, a DNA rapid extraction kit TSP101 (Tsingke Biotechnology, Beijing, China) was used. The growth rate on PDA plates was assayed as described previously [29]. For conidiation in liquid medium, the 5-mm-diameter mycelial plug was placed into a 100-mL flask containing 50 mL of sterilized PDB, the flasks were shaken at 180 rpm at 28 °C for 4 days, and the concentration of the conidial suspension was determined using a hemocytometer. For conidiation on solid medium, the strains were maintained normally on PDA or CM agar plates at 28 °C for 3 days and then transferred to alternating dark/light (12 h/12 h) conditions to promote conidiation for an additional 3–5 days. The conidial masses were observed under a stereomicroscope. For sexual reproduction, the strains were inoculated on PDA plates made with commercial PDA powder (AOBOX, Beijing, China) and cultured at 28 °C for three weeks.

### 2.2. Generation of the Cfmk1 Deletion Mutants

A split-marker approach [30] was employed for generating the *CfMK1* gene replacement cassette, as shown in schematic diagram Figure S2A. In brief, the 1040-bp upstream and 991-bp downstream flanking fragments of *CfMK1* were amplified using the primer pairs MK1/1F-MK1/2R and MK1/3F-MK1/4R (Table S1), respectively. The resulting PCR products were connected to hygromycin phosphotransferase (hph) fragments amplified with primers HYG/F-HY/R and YG/F-HYG/R (Table S1) by overlapping PCR and transformed into protoplasts of *C. fructicola* as described [29,30]. For transformant selection, hygromycin B (MDbio, China) was added to TB3 agar plate at final concentrations of 300 μg/mL. Hygromycin-resistant transformants were identified by PCR with four sets of PCR primers (MK1/5F-MK1/6R, MK1/7F-H855R, H856F-MK1/7F, and H850-H852). The sequences of the primers used for CfMK1 gene deletion and identification are listed in Table S1.

### 2.3. Generation of the CfMK1-GFP Fusion Construct

For generation of the CfMK1-GFP fusion construct, the entire CfMK1 gene, along with its promoter region, was amplified with the primers CN/F and C/R (Table S1) and cloned into *Xho*I-digested pFL2 (carrying G418 resistance marker) as previously described [31]. The CfMK1-GFP fusion construct was confirmed by sequencing analysis and transformed into the *Cfmk1* deletion mutant *Cfmk1-30* by PEG-mediated protoplast transformation [28]. For transformant selection, G418 (Sigma-Aldrich, Saint Louis, MO, USA) was added to the TB3 agar plate at final concentrations of 400 μg/mL. The resulting G418-resistant transformants were verified by PCR and examined for GFP signals by fluorescence microscope (Zeiss Axioscope 5).

### 2.4. Appressorium Formation, Penetration and Plant Infection Assays

Conidia harvested from 3-day-old PDB cultures were resuspended to a concentration of 5 × 10^4^ conidia/mL. Appressorium formation on artificial surfaces was measured as previously described [32,33]. Appressorial penetration and invasive hyphal development were assayed with onion epidermal cells [32]. For infection assays, a droplet (2 μL) of freshly harvested conidial suspensions (1 × 10^6^ conidia/mL) was dropped onto the obverse of wounded pear leaves (cv Cui guan) or fruit (cv Huang jin), stabbed by a toothpick [34,35]. Inoculated leaves were kept in a moist plastic box at 28 °C and monitored daily. Photographs were taken at seven and ten days after inoculation.

### 2.5. RNA-Seq Analysis

Mycelium of NC40 and Δ*Cfmk1*-30 were harvested from 10-day-old PDA cultures. Two biological replicates were prepared for each strain. Total RNA was extracted with a mirVana miRNA Isolation Kit (Ambion). Complementary DNA libraries were generated with TruSeq Stranded mRNA LTSample Prep Kit and sequenced with Illumina HiSeqTM 2500 at OE Biotech Co., Ltd. (Shanghai, China). RNA-Seq reads were mapped to the reference genome of the *C. fructicola* strain Nara gc5 [36] using hisat2 [37]. The fragments per kb per million reads (FPKM) value of each gene was calculated using cufflinks [38,39], and the read counts of each gene were obtained by htseq-count [40]. DEGs were identified using the DESeq R package [41] functions estimateSizeFactors and nbinomTest. False discovery rate (FDR) < 0.01 and fold change > 2 or fold change < 0.5 were set as the thresholds for significantly differential expression. Hierarchical cluster analysis of DEGs was performed to explore gene expression patterns. GO enrichment analysis of DEGs was performed using R based on the hypergeometric distribution. To filter out weakly expressed genes, only genes with a mean expression level of FPKM > 2 in at least one of the samples were included in the GO enrichment analysis. The raw RNA sequencing data have been deposited into the China National GeneBank Database (CNGBdb) under the accession CNP0002266.

## 3. Results

### 3.1. Identification and Deletion of CfMK1

The gene sequence of *CfMK1* (CGGC5_15219) was initially identified and obtained from the *C. fructicola* genome database (http://fungi.ensembl.org/Colletotrichum_gloeosporioides/Info/Index, accessed on 13 December 2021) using BLASTP with the *S. cerevisiae* Fus3/Kss1 amino acid sequence as a query. Alignment of the predicted CfMK1 protein sequence with those of other fungal Fus3/Kss1 orthologues revealed high similarity between these proteins (Figure S1A). Phylogenetic tree constructed based on the amino acid sequences of CfMK1 and these other proteins showed that CfMK1 shares sufficient homology with other fungal Fus3/Kss1 proteins (Figure S1B). Therefore, we designated CGGC5_15219 as the *C. fructicola CfMK1*. CfMK1 contained a protein kinase domain with a conserved ‘TEY’ phosphorylation motif (Figure S1A). To determine the function of *CfMK1* in *C. fructicola*, we generated *Cfmk1* deletion mutants using a split-marker method (Figure S2A) [30]. Putative *Cfmk1* deletion mutants were identified by PCR assays with four pairs of detection primers (Figure S2B), and from them, we randomly selected Δ*Cfmk1*-23 and Δ*Cfmk1*-30 for further analysis.

### 3.2. CfMK1 Deletion Affects Aerial Hyphal Growth

**Figure 1:**
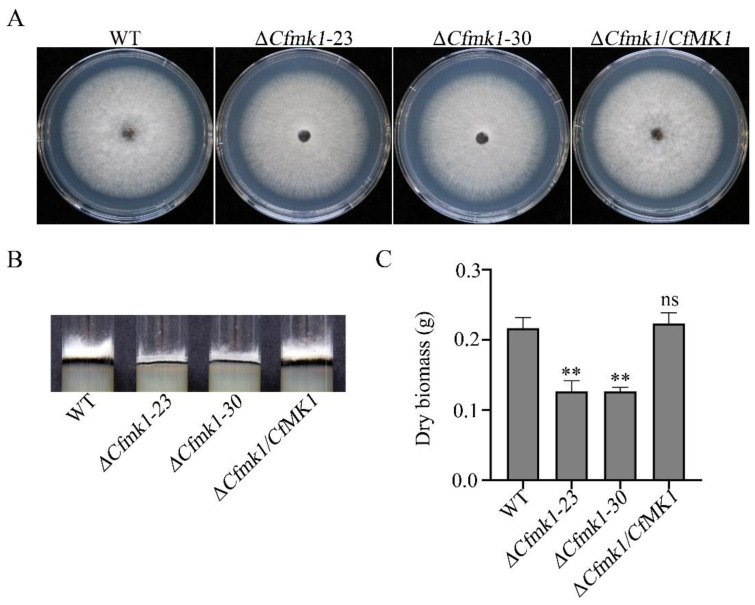
Loss of CfMK1 impairs aerial hyphal growth. (**A**) Five-day-old PDA cultures of the WT, *Cfmk1* mutants (Δ*Cfmk1-23* and Δ*Cfmk1-30*) and complemented transformant (Δ*Cfmk1*/*CfMK1*). (**B**) Aerial hyphal production of the same set of strains was observed when the strains were grown on PDA medium in test tubes. Photographs were taken after 7 days of incubation. (**C**) Dry biomass of the same set of strains after 5 days of growth on PDA plates. The error bars represent the standard deviations based on three independent biological replicates with three technical replicates each. The data were analyzed by a one-way ANOVA followed by Dunnett’s test using GraphPad Prism (version 8.0). The asterisks indicate significant differences (*p* < 0.05). ns indicate not statistically significant.

On PDA plates, the wild type (WT) strain NC40 exhibited radial growth and formed cottony colonies with substantial aerial hyphae (Figure 1A). Compared with the WT strain, the Δ*Cfmk1* mutants exhibited a normal radial colony growth rate (Figure 1A and Table 1) but formed colonies with scarce aerial hyphae clung to the surface of the PDA medium (Figure 1A,B). Likewise, the biomass production of aerial hyphae collected from PDA plates was significantly reduced (Figure 1C). These results indicate that *CfMK1* is implicated in the regulation of aerial hyphal growth.

### 3.3. CfMK1 Is Required for Asexual and Sexual Reproduction on Solid Medium but Is Not Necessary for Conidiogenesis in Liquid Medium

**Figure 2:**
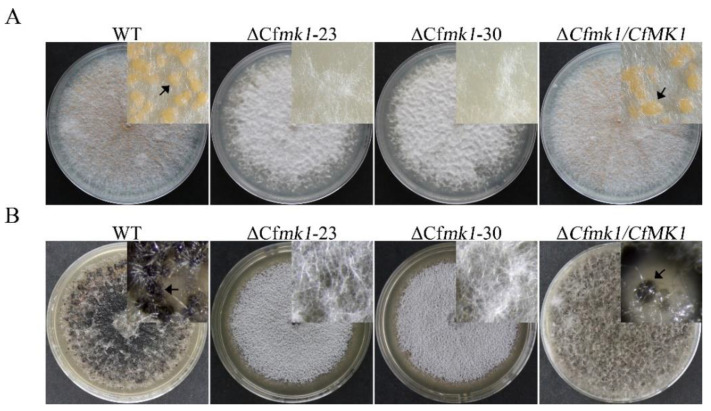
Defects of the *Cfmk1* mutant in asexual and sexual reproduction. (**A**) Conidiation of the WT, Δ*Cfmk1-23*, Δ*Cfmk1-30*, and Δ*Cfmk1*/*CfMK1* after growth on CM plates for 10 days. Conidial masses are marked with arrows. (**B**) Three-week-old mating cultures of the same set of strains. Perithecia are marked with arrows.

*C. fructicola* was able to form conidia on solid medium plates or in liquid medium incubated in a shaker. In this study, both methods were used to induce the production of conidia by *Cfmk1* deletion mutants. When cultured for 10 days on complete medium (CM) plates, both the WT and the complemented strain Δ*Cfmk1/CfMK1* formed pink conidial masses, whereas the Δ*Cfmk1* mutants did not form conidial masses (Figure 2A), indicating that CfMK1 participated in the regulation of conidiogenesis on solid medium. However, when conidiation was induced in potato dextrose broth (PDB) liquid medium by shaking, the Δ*Cfmk1* mutant produced conidia comparable to those of the WT (Table 1), indicating that CfMK1 is not necessary for conidiogenesis in liquid medium. When cultured for three weeks on PDA plates (AOBOX), both the WT and Δ*Cfmk1/CfMK1* produced black perithecia, whereas the Δ*Cfmk1* mutant did not produce perithecia (Figure 2B), indicating that CfMK1 is essential for sexual reproduction in *C. fructicola*.

### 3.4. CfMK1 Is Required for Cell Wall Integrity

**Figure 3:**
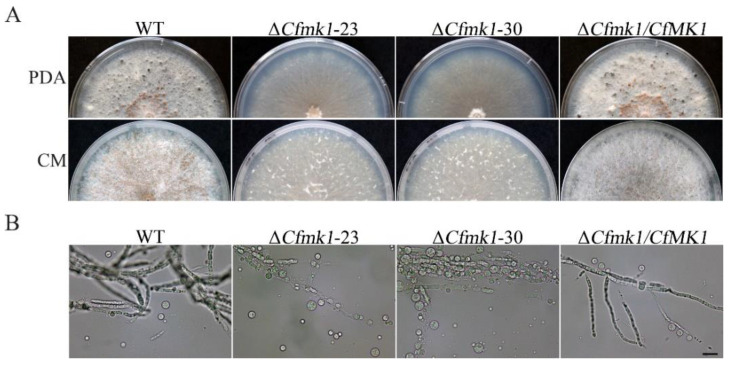
Defects in hyphal cell wall integrity of the *Cfmk1* deletion mutant. (**A**) Hyphae of the WT, Δ*Cfmk1-23*, Δ*Cfmk1-30*, and Δ*Cfmk1*/*CfMK1* cultured on PDA and CM for four weeks. (**B**) Hyphae harvested from 24-h-old PDB cultures of the same set of strains were examined after digestion with a mixture of lytic enzymes for 40 min. Bar = 20 μm.

Notably, the Δ*Cfmk1* hyphae underwent autolysis after 4 weeks of incubation on PDA plates, indicating that the cell wall integrity of the Δ*Cfmk1* mutant was defective (Figure 3A). To confirm this observation, the same set of strains were cultured on CM plates, and similar autolysis was observed 4 weeks post inoculation (Figure 3A). In addition, the sensitivity of vegetative hyphae of the Δ*Cfmk1* mutant to cell wall-degrading enzymes was tested. While the Δ*Cfmk1* mutant hyphae were almost fully digested and released a substantial volume of protoplasm after incubation with 5 mg/mL lytic enzymes for 50 min, most of the WT hyphae maintained a filamentous shape and produced fewer spheroplasts (Figure 3B), indicating that the Δ*Cfmk1* mutant had increased sensitivity to lytic enzymes.

### 3.5. CfMK1 Is Required for Conidial Germination and Appressorium Formation

**Figure 4:**
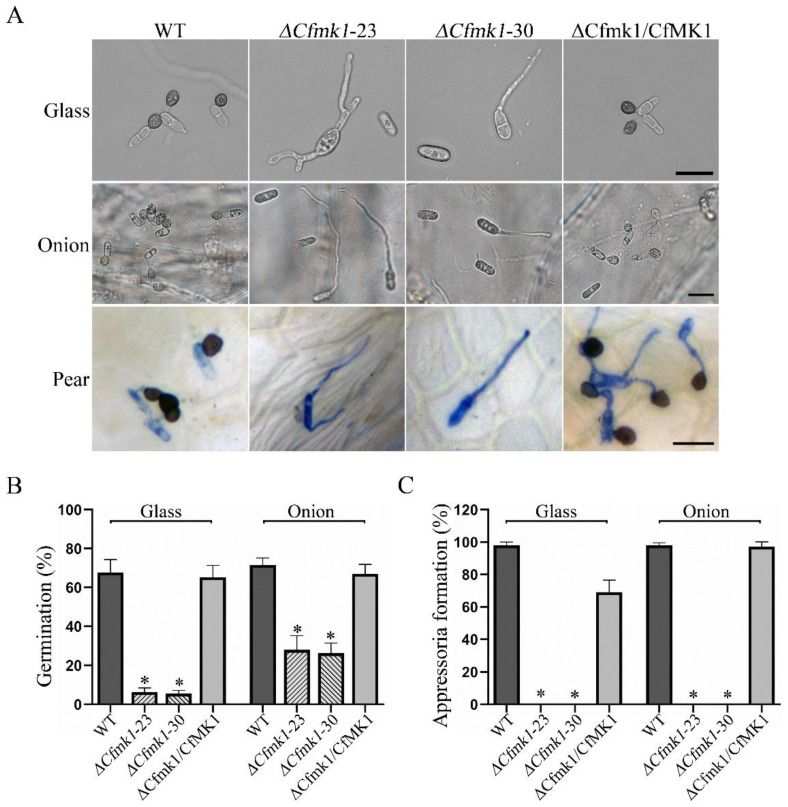
Germination and appressorium formation assays with the *Cfmk1* deletion mutant. (**A**) Conidia of the WT, Δ*Cfmk1-23*, Δ*Cfmk1-30*, and Δ*Cfmk1*/*CfMK1* were incubated on hydrophobic glass coverslips (upper panels), onion epidermal cells (middle panel), and detached pear leaves (lower panel) for 24 h. Bars = 20 μm. (**B**) Bar chart showing the conidial germination rate on hydrophobic glass slides and onion epidermal cells. (**C**) Bar chart showing the percentage of germ tubes that formed appressoria on hydrophobic glass slides and onion epidermal cells. The data were analyzed by a one-way ANOVA followed by Dunnett’s test using GraphPad Prism (version 8.0). Asterisks indicate statistically significant differences at *p* = 0.05.

To examine the role of CfMK1 in conidial germination and appressorium formation, we performed experiments on hydrophobic glass coverslips and onion epidermal cells. While 67.7 ± 6.6% of WT conidia germinated and 97.9 ± 2.2% of the germinated conidia formed appressoria after incubation for 24 h on coverslips, germination was observed in only 6.2 ± 2.1% (Δ*Cfmk1*-23) or 5.5 ± 1.7% (Δ*Cfmk1*-30) of the mutant conidia, and no appressorium was observed in the Δ*Cfmk1* mutant under the same conditions (Figure 4). When the strains were incubated on onion epidermal cells, similar results were obtained. While 71.5 ± 3.6% of WT conidia germinated and 98.0 ± 1.6% of the germinated conidia formed appressoria by 24 h post inoculation (hpi), only 27.9 ± 7.3% (Δ*Cfmk1*-23) or 26.2 ± 5.3% (Δ*Cfmk1*-30) of the mutant conidia germinated, and no appressoria were formed by the Δ*Cfmk1* mutant under the same conditions (Figure 4). When inoculated on pear leaves, while the WT and Δ*Cfmk1*/*CfMK1* conidia formed appressoria after 48 h, only germ tubes were formed by the Δ*Cfmk1* mutant (Figure 4). These results indicated that the *Cfmk1* deletion mutant had a significantly reduced conidial germination rate and was completely blocked in appressorium formation.

### 3.6. CfMK1 Is Essential for Plant Penetration

**Figure 5:**
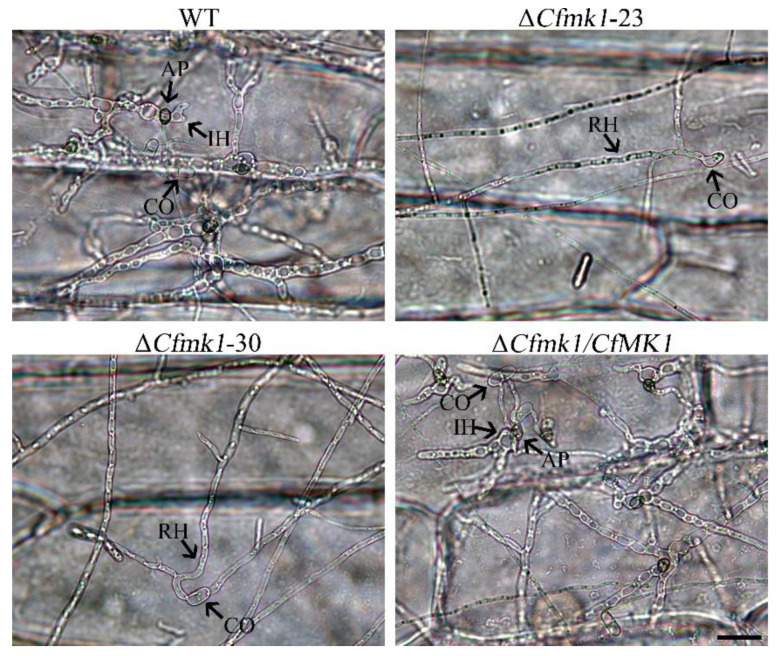
Penetration assays with onion epidermal cells. Extensive invasive hyphae were developed by the WT and Δ*Cfmk1*/*CfMK1* by 48 hpi. The tenuous running hyphae formed by Δ*Cfmk1-23* and Δ*Cfmk1-30* failed to penetrate cells, and no invasive hyphae were observed. CO, conidium; AP, appressorium; IH, invasive hypha; and RH, running hypha. Bars = 10 μm.

When examining the ability of the strains to penetrate plant epidermal cells, the WT and Δ*Cfmk1/CfMK1* appressoria penetrated onion epidermal cells and formed swollen invasive hyphae. As shown in Figure 5, the WT and Δ*Cfmk1/CfMK1* invasive hyphae had spread from the penetrated onion cells to neighboring cells by 48 hpi, whereas only running hyphae were observed outside the onion epidermal cells inoculated with Δ*Cfmk1-23* and Δ*Cfmk1-30* conidia. These results indicate that the *Cfmk1* deletion mutants could not penetrate the epidermal cells and absorb nutrition from plant tissue.

### 3.7. CfMK1 Is Necessary for Full Virulence on Pear Leaves and Fruit

**Figure 6:**
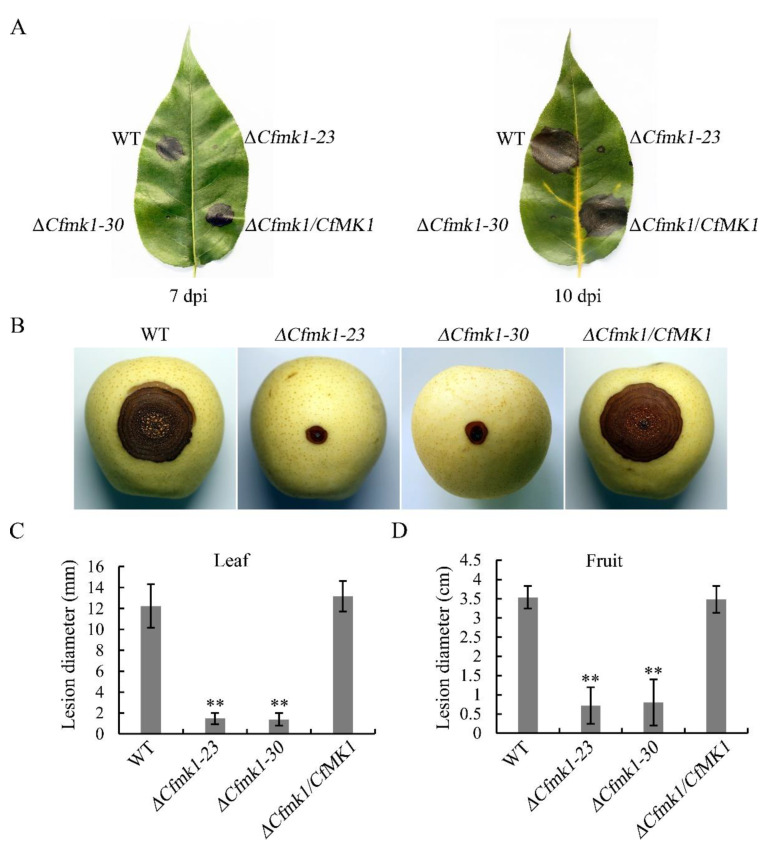
Pathogenicity assay on pear leaves and fruit. (**A**) Detached pear leaves were inoculated with conidial suspensions of the WT, Δ*Cfmk1-23*, Δ*Cfmk1-30*, and *Cfmk1*/*CfMK1*. Typical leaves were photographed 7 and 10 dpi. (**B**) The same set of conidial suspensions was inoculated into the wounded fruit. Diseased fruit were photographed at 5 dpi. (**C**) Lesion diameter of anthracnose on leaves. (**D**) Lesion diameter of anthracnose in fruit. Vertical bars represent standard deviations of the means. Asterisks indicate significant differences in the values at *p* ≤ 0.01 (**).

To examine the role of *CfMK1* in virulence, we performed pathogenicity tests by inoculating conidial suspensions onto detached pear leaves and fruit (Figure 6A,B). At 7 days post inoculation (dpi), typical anthracnose lesions were observed on leaves inoculated with the WT (Figure 6A). Under the same conditions, the *Cfmk1* mutant caused only the formation of small black spots at the inoculation sites. Even after prolonged incubation at 10 dpi, the black spots caused by the mutant remained limited to a small region around the inoculation sites (Figure 6A). On leaves, the average lesion diameter of the *Cfmk1* mutant was less than 2 mm, while the lesion diameter of the WT was more than 12 mm (Figure 6C and Table 1). Similar pathogenicity defects of the *Cfmk1* mutant were also found in infection assays with pear fruit. The *Cfmk1* mutant caused a small lesion at the inoculation sites with an average lesion diameter less than 1 cm, whereas large brown anthracnose rot with an average lesion diameter larger than 3.5 cm was observed on fruit inoculated with WT conidia (Figure 6B and Table 1). These results confirm that CfMK1 is important for virulence in *C. fructicola*.

### 3.8. Complementation and Localization of CfMK1

**Figure 7:**
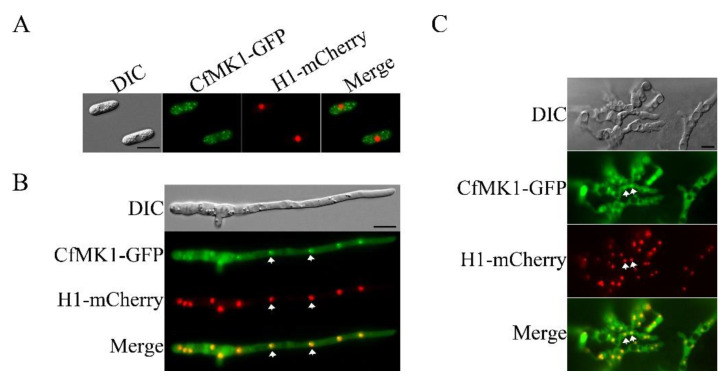
Expression and localization of CfMK1-GFP. Conidia (**A**), germ tube (**B**), and invasive hyphae (**C**) of the Δ*Cfmk1*/*CfMK1*-GFP transformant expressing the CfMK1-GFP and H1-mCherry fusion constructs. Fluorescent nuclei are marked with arrows. Bars = 10 μm.

For the genetic complementation assays, we constructed a CfMK1-GFP fusion vector using a yeast gap repair approach [31] and transformed it into the Δ*Cfmk1-30* mutant. The resulting Δ*Cfmk1*/*CfMK1*-GFP transformant exhibited normal aerial hyphal growth (Figure 1), conidiation (Figure 2), conidial germination (Figure 4), appressorium formation (Figure 4), penetration (Figure 5), and plant infection (Figure 6). These results indicated that knockout of *CfMK1* was directly responsible for the defects of the knockout mutant. When examined for the subcellular localization of CfMK1-GFP, the GFP fluorescence signals were distributed throughout the cytoplasm, and the GFP signals appeared to be enhanced at the nucleus in the germ tube and invasive hyphae (Figure 7). To confirm this observation, we cotransformed the *H1-mCherry* and *CfMK1-GFP* fusion constructs into the Δ*Cfmk1-30* deletion mutant. H1 encodes a histone protein that localizes to the nucleus in *Colletotrichum* [42]. In the resulting transformants, colocalization of H1-mCherry with CfMK1-GFP to the nucleus was observed in germ tubes and invasive hyphae, but not in conidia (Figure 7). These results indicated that CfMK1 mainly localized in cytoplasm, but may enter the nucleus during vegetative growth and invasive growth.

### 3.9. CfMK1 Deletion Affects the Expression of Different Subsets of Genes in C. fructicola

**Figure 8:**
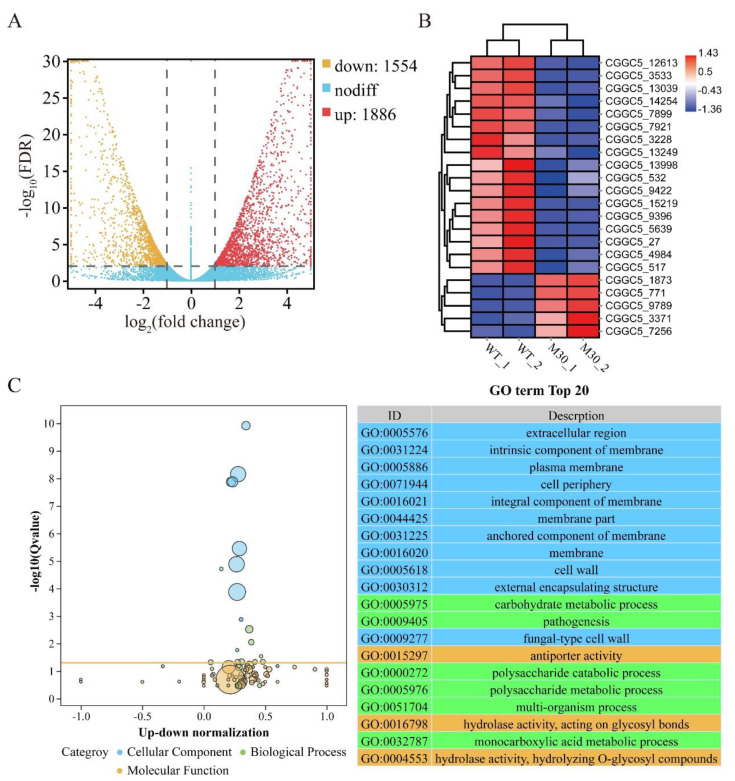
RNA-seq analysis of the Δ*Cfmk1* mutant and WT strains. (**A**) Volcano plot of DEGs between the *Cfmk1* mutant and WT. (**B**) Heat map showing the expression levels of 22 DEGs annotated to the MAPK signaling pathway. The color bars represent the values of log2-fold change (*Cfmk1* mutant vs. WT). (**C**) Significant GO analysis of DEGs. The top 20 terms are listed.

To identify genes regulated by *CfMK1* in *C. fructicola*, we performed RNA-seq analysis with RNA samples isolated from mycelium of WT and the Δ*Cfmk1*-30 mutant. Clean data were mapped to the *C. fructicola* reference genome. Unsupervised hierarchical clustering of the RNA-seq data showed that the Δ*Cfmk1* mutant mycelia segregated distinctly from the WT mycelia, indicating that deletion of the CfMK1 gene induced significant changes in the transcriptome. A total of 3440 differentially expressed genes (DEGs) were identified with significant expression differences between the Δ*Cfmk1*-30 mutant and control groups (*p*-adj < 0.01, fold change ≥ 2) (Table S2). Among them, 1554 genes were downregulated, and 1886 genes were upregulated (Figure 8A). To verify the effect of *CfMK1* deletion on the MAPK signaling pathway, we performed cluster analysis on the expression level of DEGs annotated to the MAPK signaling pathway. Among all 22 DEGs annotated to the MAPK signaling pathway, 17 DEGs were downregulated in the Δ*Cfmk1* mutant (Figure 8B, Table S3). Notably, two key components in the Ste11-Ste7-Fus3 MAPK cascades, namely, CGGC5_517 (homology to Ste11) and CGGC5_9396 (homology to Ste7), were downregulated significantly in the Δ*Cfmk1* mutant, indicating that the Fus3/Kss1 pathway has a feedback regulation mechanism. In addition, the expression level of CGGC5_13039 (homologous to Slt2), the central kinase in the Bck1-Mkk1/Mkk2-Slt2 cascades, was also downregulated significantly, suggesting that there may be crosstalk between the Fus3 and Slt2 MAPK signaling pathways. Then, analysis of significant Gene Ontology (GO) terms was performed, and the top 20 GO terms are listed (Figure 8C). In the cellular component category, most of the DEGs were significantly enriched in extracellular region, membrane, and cell wall terms, which correspond to the defects in the cell wall integrity of the *Cfmk1* mutant. In the biological process category, carbohydrate metabolic processes changed most significantly, followed by pathogenic processes. In the molecular function category, antiporter activity changed most significantly, followed by hydrolase activity, acting on glycosyl bonds.

## 4. Discussion

The FUS3/KSS1-type MAPK CfMK1 described in this study belongs to the fungal extracellular signal-regulated kinase subfamily of the MAPK superfamily. In *S. cerevisiae*, Fus3/Kss1 is the final kinase in the signal transduction module regulating mating, filamentation and invasion [43]. In phytopathogenic fungi, Fus3 MAPK is important for disease development. This MAPK is essential for appressorium formation in all appressorium-forming pathogens studied, including *M. oryzae*, *B. cinerea*, and *C. gloeosporioides*, and plays important roles in plant penetration and invasive growth in various non-appressorium-forming pathogens [19]. In this study, we found that the *Cfmk1* deletion mutant exhibited blocked appressorium formation, was defective in plant penetration and invasive growth, and exhibited significantly reduced virulence in pear leaves and fruit, supporting the viewpoint that this MAPK pathway is widely conserved in pathogenic fungi for regulating infection processes. Recently, RNA interference-based fungicides have been recognized for their potential applications in crop protection. The Fus3 homolog in *Puccinia triticina* was used as a promising target to control rust disease, and transgenic wheat harboring a Pmk1-RNAi vector showed significant resistance to the disease [44]. In the future, CfMK1 can potentially be targeted for broad-spectrum pear anthracnose control, overcoming the challenges posed by the complexity of the species composition of pear anthracnose causal agents.

The fungal cell wall is a basic structure with high plasticity that is essential for maintaining cell integrity and vitality [45]. In budding yeast, cell wall integrity is regulated by the Slt2 MAPK signaling pathway. In this study, we found that deletion of *CfMK1* resulted in poorly developed aerial hyphae and apparent hyphal autolysis, which is similar to the defects exhibited by deletion mutants of genes in the CgMK1 MAPK cascade in *C. gloeosporioides* [46]. In addition, the *Cfmk1* deletion mutant was more sensitive to cell wall lysozyme than the WT. These results suggest that CfMK1 is involved in the regulation of cell wall integrity. In *F. graminearum*, overactivation of Gpmk1 MAPK affected genes related to cell wall integrity [47]. In *C. higginsianum*, a Chmk1 deletion mutant was hypersensitive to cell wall inhibitors [23]. Although the Slt2 pathway has been considered the main signaling pathway responsible for cell wall integrity, other signaling pathways have also been implicated in maintaining cell wall structure [47,48]. According to our RNA-seq results, the expression of the kinase Slt2 was downregulated in the *cfmk1* deletion mutant. These results suggest that there may be crosstalk between the Fus3 pathway and Slt2 pathway. Therefore, it will be important to characterize the role of CfMK1 in cell wall biosynthesis and its relationship with Slt2 in *C. fructicola*. The synthesis and maintenance of the cell wall involves a large number of biosynthetic pathways [45]. GO enrichment analysis in this study revealed that cell wall- and fungal-type cell wall terms were enriched with more than 50 DEGs, including three chitin synthase genes, namely, chs3 (CGGC5_1611), chs8 (CGGC5_13604), and chsD (CGGC5_2057), and two chitin synthase activators, namely, chr4 (CGGC5_12645) and skt5 (CGGC5_7100). These results suggest that the defects of the cell wall in the *cfmk1* deletion mutant may be partially attributed to the disruption of chitin synthesis.

Fungi possess various specialized physiological and developmental strategies for dispersal, reproduction, and long-term survival [49]. Ascomycetes produce asexual spores, called conidia, when environmental conditions are no longer suitable for growth by apical extension [50]. In *M. oryzae* and *B. cinerea*, deletion of the *Fus3* homolog did not affect conidiation [12,51], while in *C. lagenarium*, *C. truncatum*, *C. higginsianum*, and *F. graminearum*, conidiation was significantly reduced [23,24,25,52]. In this study, the *Cfmk1* deletion mutant exhibited significantly reduced conidiation on solid medium plates, consistent with the results for other *Colletotrichum* species. Notably, in previous studies, conidiation was assayed on solid medium. The conidiation of *Colletotrichum* can be induced both on solid medium and in liquid medium [53]. Interestingly, when conidiation was induced in liquid medium, the *Cfmk1* mutant produced an equivalent quantity of conidia as the WT strain. Thus, we speculate that conidiation on solid medium is a MAPK-regulated process, while conidiation in liquid medium is a CfMK1-independent process, in *C. fructicola*. In addition, we found that the conidial germination rate of the *Cfmk1* knockout mutant was substantially reduced, indicating that CfMK1 was essential for conidial germination. In *C. lagenarium*, conidia of *cmk1* mutants fail to germinate on both host plant and glass surfaces [52], whereas *C. truncatum* conidia from the *Ctpmk1* mutant germinate normally on glass slides and onion epidermal surfaces [24]. These results suggest that Fus3-type MAPKs might have distinct roles in conidiogenesis and conidial germination among filamentous fungi. Since conidia are the main overwintering forms in the field, their production and germination ability are essential for the spread and prevalence of pear anthracnose; thus, we speculate that *CfMK1* is important for the survival of *C. fructicola* in nature.

Pathogenesis is a very complex process, involving a variety of signaling pathways, gene regulatory relationships and other processes. In this study, infection assays showed that the *Cfmk1* mutant exhibited significantly reduced pathogenicity. The defects of the *Cfmk1* mutant in plant infection may be related directly to its defects in conidial germination, appressorium formation and penetration of the plant epidermis. However, the Cfmk1 deletion mutants were also defective in wound infection, suggesting a vital role of CfMk1 during invasive growth. In *M. oryzae*, Pmk1 was reported to control the hyphal constriction required for fungal spread from one rice cell to the neighboring cell, which is important for host tissue colonization [54]. A similar regulatory mechanism may exist in *C. fructicola*. In addition, 62 DEGs were significantly enriched in pathogenesis-related terms (Table S4), including the C6 finger domain-containing protein CGGC5_3069, the cutinase-encoding gene CGGC5_1134, and histidine kinase protein CGGC5_4992. In *M. oryzae*, Pmk1 was reported to positively regulate transcription factors such as Mst12, Mcm1 and Sfl1 during different infection processes [19]. CGGC5_3069 may be a new transcription factor regulated by CfMk1, but this needs further verification. Cutinase is an extracellular degradative enzyme produced by plant pathogenic fungi for degrading the plant cuticle layer and plays an important role in penetration by pathogens and carbon acquisition [55]. The cutinase gene CglCUT1 is required for the pathogenicity of *C. gloeosporioides,* causing anthracnose of *Camellia oleifera* [56]. The histidine kinase MoPAS1 (homolog of CGGC5_4992) has been reported to be important for conidiogenesis, appressorium formation, and pathogenesis in *M. oryzae* [57]. Downregulated expression of CGGC5_1134 and CGGC5_4992 in the *Cfmk1* mutant may be part of the reason for the decline in pathogenicity.

In summary, we found the MAPK gene *CfMK1* plays important roles in regulating aerial hyphal growth, sexual reproduction, appressorium formation, plant penetration, and pathogenicity in *C. fructicola*. In this study, we demonstrated that the Fus3/Kss1-related MAPK pathway might be involved in conidiogenesis on solid medium, but not in liquid medium. Our study also showed that CfMK1 was localized in the cytoplasm of conidia, germ tubes, and invasive hyphae and enriched in the nucleus during vegetative growth and invasive growth but not in the conidial stage. Moreover, we identified 3440 differentially expressed genes in the *Cfmk1* mutant compared with the WT and revealed that CfMK1 might be involved in regulating fungal cell wall integrity and pathogenesis by affecting the expression of these process-related genes.

## Figures and Tables

**Table 1:** Defects of the Δ*Cfmk1* mutant in growth, conidiation, and plant infection.

Strains	Growth Rate (mm/d) ^1^	Conidiation (10^5^/^mL^) ^2^	Disease Diameter (mm) ^3^	
			Leaf	Fruit
NC40 (WT)	6.1 ± 0.1 ^a^	3.2 ± 0.6 ^a^	12.2 ± 2.1 ^a^	35.4 ± 3.0 ^a^
Δ*Cfmk1*-23	5.9 ± 0.1 ^a^	3.7 ± 0.8 ^a^	1.5 ± 0.5 ^b^	7.2 ± 4.8 ^b^
Δ*Cfmk1*-30	5.9 ± 0.0 ^a^	3.3 ± 0.7 ^a^	1.4 ± 0.6 ^b^	8.0 ± 6.0 ^b^
Δ*Cfmk1/CfMK1*	6.2 ± 0.1 ^a^	3.2 ± 0.5 ^a^	13.2 ± 1.5 ^a^	34.8 ± 3.5 ^a^

^1^ Average daily extension in colony radius on PDA plates. ^2^ Conidiation in 3-day-old PDB cultures. ^3^ Disease diameter measured on inoculated leaves (7 dpi) and fruit (5 dpi). Means and standard deviations were calculated from three independent measurements. The data were analyzed with Fisher’s protected least significant difference (LSD) test. Different letters within the same column indicate significant differences (*p* = 0.05).

## Data Availability

The raw RNA sequencing data have been deposited into China National GeneBank Database (CNGBdb) under the accession CNP0002266.

## Supplementary Materials

The following are available online at https://www.mdpi.com/article/10.3390/jof8010077/s1: Figure S1, Characterization of the MAPK CfMK1 in *C. fructicola*; Figure S2, Generation of *CfMK1* gene deletion mutants; Table S1, PCR primers used in this study; Table S2, Expression level of 3440 DEGs identified between the *Cfmk1* mutant and WT; Table S3, Expression level of 22 DEGs annotated to the MAPK signaling pathway; and Table S4, Expression level of 62 DEGs enriched in pathogenesis terms.
