# Supplementary material for: The CfMK1 Gene Regulates Reproduction, Appressorium Formation, and Pathogenesis in a Pear Anthracnose-Causing Fungus
Source: J Fungi (Basel). 2022 Jan 14;8(1):77. doi: 10.3390/jof8010077 (PMC8779585; doi:10.3390/jof8010077)
Supplement: Supplementary file 1 [file jof-08-00077-s001.zip › Supporting figures.pdf]

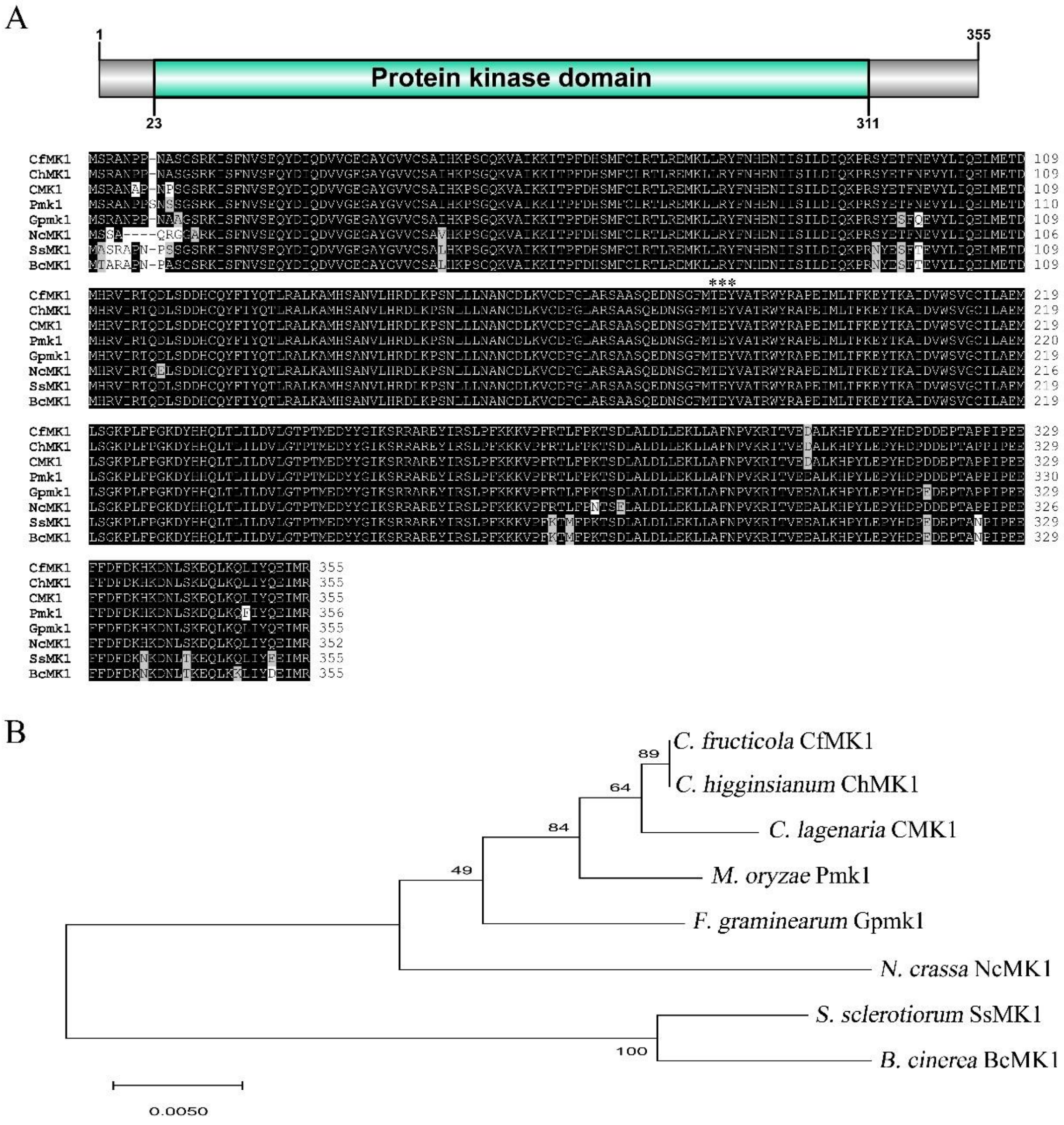

Fig. S1 Characterization of the MAPK CfMK1 in *C. fructicola*. (**A**) Domain structure and amino acid sequence alignment of CfMK1. (**B**) Phylogenetic analysis of CfMK1 and its homologs from other fungi.

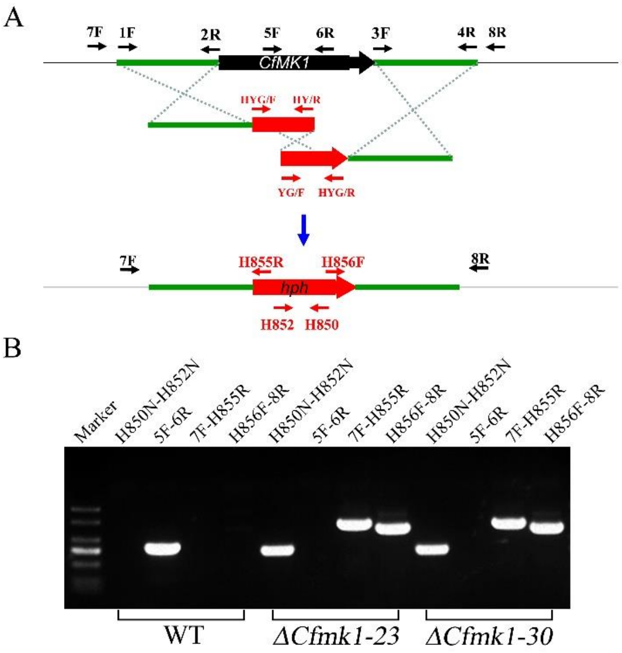

Fig. S2 Generation of *CfMK1* gene deletion mutants. (**A**) Schematic diagram of gene deletion with the split-marker approach. The primers used for gene replacement and screening are listed in Table S1. (**B**) Deletion mutants were detected by four pairs of primers (Table S1). Hph, hygromycin.
